# Building a pan-European network to bridge gaps in geriatric medicine education: the PROGRAMMING COST Action 21,122—a call for endorsement

**DOI:** 10.1007/s41999-024-01137-0

**Published:** 2025-02-19

**Authors:** Sofia Duque, Karolina Piotrowicz, Tahir Masud, Anne Wissendorff Ekdahl, Anna Marie Herghelegiu, Tajana Pavic, Evrydiki Kravvariti, Nenad Bogdanović, Sylvie Bonin-Guillaume, Nicolas Martínez Velilla, Regina Roller Wirnsberger, Michael Vassallo, Anastassia Kossioni, Rachael Frost, Jurate Macijauskiene, Meltem Koca, Athanase Benetos, Mirko Petrovic, Marina Kotsani

**Affiliations:** 1 Department of Internal Medicine, Hospital CUF Descobertas; Preventive Medicine and Public Health Institute, Faculty of Medicine, University of Lisbon, Lisbon, Portugal; 2 Department of Internal Medicine and Gerontology, Faculty of Medicine, Jagiellonian University Medical College, Jakubowskiego 2 Str., 30-688 Kraków, Poland; 3 Department of Health Care of Older People, Nottingham University Hospitals NHS Trust, Nottingham, UK; 4 Faculty of Medicine, Institution of Clinical Sciences Helsingborg, Lund University, Lund, Sweden; 5 Geriatrics and Gerontology Department – “Ana Aslan” National Institute of Gerontology and Geriatrics, “Carol Davila” University of Medicine and Pharmacy, Bucharest, Romania; 6 Department of Gastroenterology and Hepatology, School of Medicine, Sestre Milosrdnice University Hospital Center Zagreb, University of Zagreb, Zagreb, Croatia; 7 Postgraduate Medical Studies “Physiology of Aging and Geriatric Syndromes” School of Medicine, National and Kapodistrian University of Athens, Athens, Greece; 8 Division of Clinical Geriatrics, Department for Neurobiology, Caring Science and Society – NVS, Karolinska Institutet, Stockholm, Sweden; 9 Internal Medicine and Geriatric Department, University Hospital of Marseille, Aix Marseille University, Marseille, France; 10 Department of Geriatrics, Navarre Health Service (SNS-O), Navarre University Hospital (HUN), Navarrabiomed, Navarre Public University (UPNA), Navarra Institute for Health Research (IdiSNA), Pamplona, Spain; 11 Unit for Education and Training, Department of Internal Medicine, Medical University of Graz, Graz, Austria; 12 University Hospitals Dorset, Royal Bournemouth Hospital, Bournemouth University, Bournemouth, England UK; 13 Department of Prosthodontics, Dental School, National and Kapodistrian University of Athens, Athens, Greece; 14 School of Public and Allied Health, Liverpool John Moores University, Liverpool, UK; 15 Department of Geriatrics, Lithuanian University of Health Sciences, Kaunas, Lithuania; 16 Geriatrics Clinic, Turkish Ministry of Health Etlik City Hospital, Ankara, Turkey; 17 Pôle “Maladies du Vieillissement, Gérontologie et Soins Palliatifs”, INSERM DCAC u1116, Université de Lorraine, CHRU-Nancy, 54000 Nancy, France; 18 Section of Geriatrics, Department of Internal Medicine and Paediatrics, Ghent University, Ghent, Belgium; 19 Hellenic Society for the Study and Research of Aging, Athens, Greece

**Keywords:** Geriatric medicine, Healthcare education, Education and training, PROGRAMMING, Comprehensive geriatric assessment, Patient-centered care

## Abstract

**Background:**

The growing challenges of population aging create a pressing need for specialized geriatric medicine services to effectively address the complex health needs of older adults and influence supportive healthcare policies. Older patients may present clinical complexity with multimorbidity, disability, and/or frailty, necessitating a shift from the traditional organ-oriented clinical approach to a holistic, patient-centered care model.

**Rationale of the action:**

Despite recommendations from the World Health Organization and scientific and professional societies, geriatric medicine is not universally recognized as a distinct specialty in Europe, and education in this field remains heterogeneous. A notable discrepancy in the availability of geriatric services and education in this field across European countries can be found. Many healthcare professionals lack basic training in geriatric medicine, contributing to fragmented care and poorer health outcomes. To address these challenges, it is essential to integrate geriatric medicine into undergraduate and postgraduate curricula for all healthcare professionals.

**Expected outcomes:**

The COST Action 21,122 PROGRAMMING (PROmoting GeRiAtric Medicine in countries where it is still eMergING) initiative aims to promote geriatric medicine by developing targeted educational goals and programs and fostering interdisciplinary collaboration. This initiative aims to assess the current state of geriatric medicine education and identify both global and local educational needs for developing clinical skills among healthcare professionals. In addition, it seeks to establish consensus on core curricula tailored to local contexts and disseminate findings and recommendations to stakeholders, policymakers, and the public. By uniting diverse stakeholders, PROGRAMMING aspires to create sustainable changes in geriatric care across Europe.

**Graphical abstract:**

## Key summary points

**Aim:**

To advocate for collaborative efforts to promote the advance of Geriatric Medicine through interdisciplinary teamwork and educational activities, with the establishment of core geriatric principles in the curricula of different disciplines to address the complex health needs of older adults.

**Findings:**

Geriatric Medicine remains underrepresented as a specialty in many European countries, with healthcare professionals lacking proper training and practical skills, leading to fragmented and suboptimal care for older adults. The COST Action 21122 PROGRAMMING initiative aims to enhance education and foster international collaboration to address these gaps and improve healthcare outcomes for older adults.

**Message:**

Engaging a broad network of endorsing members and organizations will enhance the impact of the PROGRAMMING CA 21122, fostering comprehensive Geriatric Medicine development with targeted education and training across Europe.

## Graphical abstract

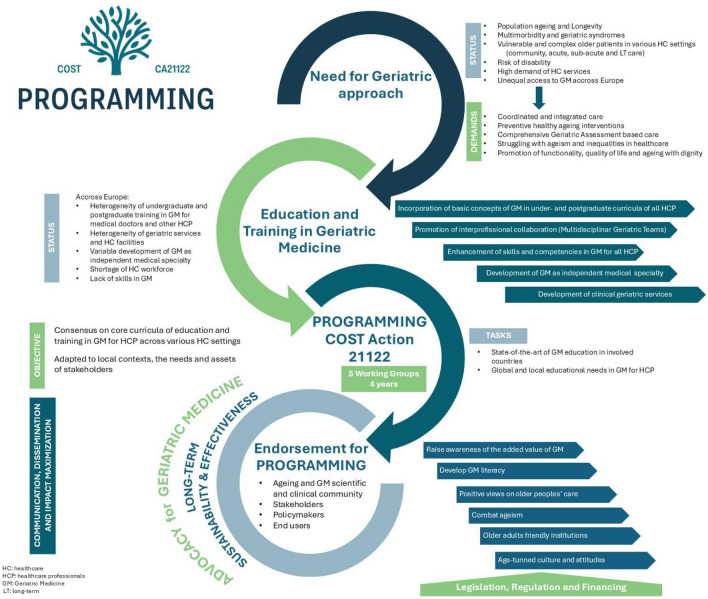

## Introduction

In 2019, the European Geriatric Medicine Society (EuGMS), with the mission of fostering geriatric medicine across Europe, launched the Global Europe Initiative (GEI), a group designated to reinforce its actions by spreading educational activities and high professional standards in geriatric medicine across Europe. A greater focus was given to countries where geriatric medicine is still emerging or has yet to obtain professional and scientific independence and recognition. Despite the significant heterogeneity of geriatric care models across different countries, certain minimum requisites are essential for appropriate geriatric care. These include comprehensively assessing older patients to deliver personalized and meaningful interventions that promote functionality and quality of life, ensuring the smooth integration of health and social care, and providing education on the basic principles of geriatric medicine in a transversal manner to healthcare professionals, older adults, and caregivers.

The major goal of the GEI is to promote the development of geriatrics locally. Since the beginning, promoting joint actions and educational activities, such as on-site and online meetings, has been one of the main actions to establish connections and collaborations between members coming from countries where geriatric medicine is still emerging [1]. In this context, the idea of submitting a proposal to the European Cooperation in Science and Technology (COST) emerged and the GEI led the preparation of the application and the raising of the initial group of proposers, to which other EuGMS groups, such as the Early Career Geriatricians’ Initiative and the “Education and Training” and “Gerodontology” Special Interest Groups contributed, as well as the European Academy for the Medicine of Ageing (EAMA). The proposal was approved for funding and PROGRAMMING- PROmoting GeRiAtric Medicine in countries where it is still eMergING COST Action 21,122 kicked off on November 2nd, 2022 [2].

PROGRAMMING’s mission is to identify a pragmatic set of possibilities for continuous professional education in geriatric medicine, tailored to non-specialist professionals and adapted to local contexts, the needs and assets of stakeholders and the pragmatic possibilities of involved settings. PROGRAMMING outputs, expected to be globally endorsed, will be proposed to stakeholders and policymakers across Europe, to encourage change at the national level, promoting the integration of key geriatric medicine principles into practitioners’ attitudes, practices, and services [3].

To be effective in this mission, PROGRAMMING’s main messages must be supported and endorsed by leading societies, scientific and academic bodies in geriatric medicine and allied healthcare disciplines, as well as by policymakers and key stakeholders. To strengthen our message and facilitate its advocacy, dissemination, and impact maximization, we publish this call to endorse document (Table 1).

**Table 1:** PROGRAMMING CA 21122 endorsement statements

Statement 1: There is an urgent need for geriatrics training for healthcare professionals caring for older patients. Establishing geriatric medicine as a distinct specialty across Europe is a key factor in enhancing education and healthcare services tailored to older adults
Statement 2: To meet the growing demand for geriatric expertise, both geriatricians and other trained healthcare professionals must collaborate in caring for older adults. Incorporating geriatric medicine into all healthcare curricula is a priority
Statement 3: Raising awareness about the added value of geriatric medicine among stakeholders, including the public and older adults, is crucial. This will foster advocacy and engage policymakers to establish sustainable geriatric medicine services across Europe and beyond

## The need for a geriatric approach

The population aging and the high burden of acute and chronic diseases of older persons will increase the demand for healthcare services and the need for specialized care for older persons, both at community and acute or sub-acute care and long-term care settings [4, 5]. Most healthcare users are older persons, and consequently most healthcare professionals will likely interact with older patients in their daily clinical practice [6].

Older patients often experience multimorbidity and related polypharmacy, disability and/or frailty, and present with atypical presentation of illnesses, multiple drug–drug and drug–disease interactions, a higher likelihood of drug-related adverse effects and other iatrogenic complications, and the overlap between chronic conditions and geriatric syndromes. Geriatric medicine is a medical discipline specializing in the care of vulnerable and complex multimorbid older patients and plays a crucial role in supporting and promoting healthy aging. Although aging is a natural and inevitable process, aging trajectories can vary widely—from successful aging, where functional ability and autonomy are preserved, to frailty, disability, and dependency at the other extreme. Geriatric medicine is uniquely positioned to anticipate these diverse trajectories and to implement preventive interventions that mitigate both the physiological effects of aging and the pathophysiological mechanisms underlying chronic conditions [6].

The conventional organ/disease-oriented clinical approach may result in older patients with complex multimorbidity to accumulate injudicious diagnostic and therapeutic interventions and medication as various specialists manage their conditions simultaneously but usually independently, each one dealing with a single disease [7, 8]. This type of fragmented care is even more prevalent in countries where geriatric medicine is underdeveloped or not well integrated into the healthcare system, resulting in services that are insufficient to meet the needs of older adults [9]. Moreover, many older patients do not fit in a single-disease management algorithm [10], and strict single-disease-oriented applications of guidelines to multimorbid older patients can hinder health-related outcomes, quality of life, and survival [11, 12]. On the other hand, relevant interventions to promote healthy aging and optimize functionality, such as healthy nutrition, physical activity, and vaccination, are frequently omitted in plans of care. Moreover, geriatric syndromes, although major causes of disability, often remain unrecognized and thus undertreated [13].

The lack of access to geriatric medicine for older patients in various countries across Europe, regardless of the underlying causes, can be seen as a form of ageism, as they are deprived of appropriate medical care. This situation not only highlights a significant injustice but also underscores the need for inclusion, demanding that efforts be made to eliminate these health inequalities in the Europe. Notably, the United Nations has recently addressed the urgency of expanding training and educational opportunities in geriatrics and gerontology to promote aging with dignity [14].

In recognition of the impact of geriatric medicine, there has been a growing demand by various medical specialties for a more tailored approach to the older patient to enhance outcomes. This collaborative effort has been noted specifically in areas such as oncogeriatrics, orthogeriatrics, and cardiogeriatrics [15–17], and expands to other healthcare disciplines such as gerodontology or geriatric nursing.

The Comprehensive Geriatric Assessment (GCA) is the most valuable tool that geriatric medicine utilizes to approach the complexity of the older patient, especially one living with frailty, and proposes a patient-tailored plan of care. The CGA is a multidimensional diagnostic approach, which often relies on interdisciplinary teams, and differs from a standard medical evaluation by including non-medical domains, by emphasizing functional ability, quality of life, patient priorities, preferences, and patient-relevant goals [18, 19]. Although the CGA requires specific infrastructure and, most importantly, specialized human resources, its cost-effectiveness and comprehensive approach to the health, functionality, and well-being of older adults justify it becoming the standard of care for this population [20]. The cost-effectiveness of the CGA, the standard methodology in geriatric medicine, is a crucial aspect to advocate for among policymakers if we intend for them to incorporate it structurally into healthcare systems. Despite some conflicting results due to the heterogeneity of the CGA, variations in healthcare systems, and indirect costs (such as those related to social needs) [21], there is data supporting the cost-effectiveness of the CGA across different healthcare settings, including hospitals, ambulatory care, and primary care. Studies on CGA-based interventions have shown gains in quality-adjusted life years (QALYs), cost savings, or a fair expenditure for the benefits gained [20, 22, 23]. Overall, the CGA can be considered cost-effective for selected vulnerable populations in various settings, and it is essential for policymakers to incorporate it into healthcare policies to enhance care for older adults.

The World Report on Ageing and Health that the World Health Organization (WHO), released in 2015, claims for coordinated inter-professional care to ensure safety and effectiveness at different levels of care [24]. It supports aspects such as the shift from a disease-oriented to a person-oriented approach, the need to integrate health and social care, the prevention and management of age-related conditions, the promotion of healthy aging and the importance of respecting patients’ needs and preferences [24, 25]. A CGA performed by multidisciplinary teams is the evidence-based instrument to implement the inter-professional collaborative practice such as supported by the WHO. These teams speak a common language and refer to the same principles [26, 27]. The WHO has operationalized these needs by designing the Integrated Care for Older People (ICOPE) pathway, which aligns closely with the CGA approach. It is a person-centered strategy focused on delivering preventive and personalized care plans that address some domains of the CGA. ICOPE contributes to the development of essential geriatric competencies and skills among healthcare professionals dealing with older adults in community settings [28].

Moreover, among the sustainable development goals of the Decade of Healthy Ageing 2021–2030 promoted by the United Nations, the integrated care and accessibility to long-term care are identified as two enablers to reach healthy aging and are both endeavors closely related to geriatric medicine [29].

The above-mentioned aspects are in perfect alignment with the principles of geriatric medicine, and they should be embraced by all healthcare professionals dealing with older patients, extending beyond just geriatricians and geriatric teams [30]. This highlights the need for all healthcare professionals to acquire a minimum set of knowledge and skills for the prevention and management of age-related health conditions and iatrogenic complications and the orientation of healthcare personnel’s attitudes and practices toward a patient-centered integrated care for the older person [28].

## Education and training in geriatric medicine: a feasible approach to improve older persons’ care

Despite the recommendations and sustained efforts of organizations such as the WHO and professional and scientific societies such as the EuGMS to enhance care for older individuals, there is currently significant variation in the availability of specialized health care structures, services, and geriatric education across Europe [9, 31–33]. National specificities and differing policymaker priorities in different European countries contribute to this heterogeneity. Challenged with a rising number of older patients and associated caregivers, along with their increasingly complex needs, the situation of healthcare and social services is further complicated by the absence of standardized training in older patient care for specialized medical staff [34–36].

Besides the fact that the growing shortage of healthcare professionals is a major challenge in many European countries, this is particularly the case for healthcare workforce with specific training and skills for caring for older people [37]. Career choices of health professionals reflect a declining attraction toward certain medical specialties often perceived by students as too difficult, demanding excessive professional commitment or lacking in prestige and sufficient remuneration in some countries, and geriatric medicine is among these specialties facing a lack of interest [38].

In addition to the problem of attractiveness, and despite the availability of European recommendations for the postgraduate curriculum in geriatrics as a medical specialty [39], according to publicly available data geriatric medicine was recognized as an independent medical specialty in only 23 European countries out of 53^1^ [1, 33, 40]. Being recognized as a specialty (or subspecialty in some cases) does not guarantee that geriatric medicine is fully developed, and some gaps in geriatric care may still be evident. In such cases, actions to promote the development of geriatric medicine may still be necessary. On the other hand, in some countries where geriatric medicine is not recognized as a specialty or subspecialty, high standards of geriatric care might be observed. This non-universal recognition of geriatrics across Europe is likely linked to the barriers for the development of clinical geriatric services and limits the availability of expertise in geriatric medicine [33, 41].

Moreover, despite recommendations on geriatric content having been established for undergraduate medical students [42], undergraduate training in medical schools show even less uniformity. Undergraduate medical curricula in several European countries still do not incorporate education and training in geriatric medicine as a distinct examination subject, and topics related to older adult care are fragmentated and incorporated into training programs for internal medicine, psychiatry, neurology, or other disciplines, and the training models for medical students differ significantly among countries as well [35].

Similar problems are observed in the undergraduate education of other healthcare professions caring for older patients. Studies from North America on undergraduate training on geriatric medicine of nursing, nutrition, and pharmacy students reported a deficiency in geriatrics content [43–45].

To tackle the aforementioned challenges, a thoughtful approach should involve the incorporation of basic concepts of geriatric medicine into the undergraduate and postgraduate curricula of various healthcare professionals [24, 46]. In its 2015 report, the WHO also declared that refining knowledge and skills in the care for older patients is imperative for professionals in all health disciplines [24]. Promoting education and training of all healthcare professional dealing with older people in basic principles of geriatric medicine will not only enhance competences and skills for the improvement of the quality of care of older people but will also facilitate inter-professional collaboration and a positive attitude shift regarding the care of the older patient and older persons themselves.

It is important to highlight that basic training in geriatrics for various healthcare professionals does not eliminate the need for specialized professionals in the treatment of older individuals and should not undermine the advocacy for the establishment of geriatric medicine in all European countries. Given the currently insufficient ratio of geriatricians to older citizens in Europe, it becomes imperative to wisely manage available human resources by optimizing workloads and task delegation [40]. A pragmatic strategy involves sharing of certain responsibilities and tasks between geriatricians and other healthcare professionals, after undergoing adequate training, which must be incorporated into continuous education frameworks and established as standard best practices within healthcare systems [47]. For example, these trained professionals could undertake early tasks in geriatric screenings and chronic diseases management as well as promoting deprescribing and supporting treatment. Non-physician members of the team, properly trained, should possess essential knowledge in recognizing and screening for prevalent geriatric syndromes and chronic comorbidities and be equipped with skills, tools, and methods to address them. The role of the geriatrician would involve synthetizing the results of the CGA, leading the geriatric care plan, collaborating with various healthcare professionals and other medical specialties, and coordinating interventions across the spectrum of health services. In addition, geriatricians would manage complex cases in specialized settings, train other professionals in geriatrics and contribute to research in older people with frailty [48]. Moreover, the recognition of geriatric medicine as a specialized discipline, with professionals devoted to the care of older adults, especially those with the most complex social and clinical profile, is essential to foster a professional education and culture tailored to the older patient’s needs and advocate it across the entire healthcare spectrum.

Therefore, to improve older persons’ care, alleviate inequalities across Europe and to develop healthcare services aligned to the needs of the older populations, as prioritized by the WHO (2015), key actions include both reinforcing geriatric medicine as a medical specialty in all European countries and ensuring a set of essential skills and competences in geriatrics, at different career stages, for the majority of healthcare professionals.

Medical and other allied healthcare professional education programs, as well as national plans to address the growing health needs of aging populations, depend on academic and political decisions and may take extensive time to show results.

However, there is already substantial evidence from countries with geriatric medicine services, both general and tailored for specific populations, demonstrating benefits related to healthcare usage (such as reduced length of stay, lower rates of emergency department admissions, and decreased hospital readmission rates), clinical outcomes (including improved recovery, reduced complications, reduced mortality, reduced delirium, pressure ulcers incidence, rate of falls, decline of risk of frailty), long-term care usage (such as lower nursing home admission rates), treatment management (such as rates of polypharmacy, treatment compliance, toxicity risk), patient satisfaction and quality of life, as well as benefits for caregivers (such as decreased burden and improved support) [18, 49–54].

In the meantime, providing ongoing professional education in the basic concepts of geriatric medicine to the current healthcare workforce is a practical and achievable step. In this effort, scientific societies and international networks can play a crucial role.

## PROGRAMMING COST Action 21,122: creation, concrete objective, and aspiration

The concrete objective of PROGRAMMING is to reach a consensus on the content of short, targeted education and training activities in geriatric medicine for healthcare professionals across various clinical settings. This objective is aimed mainly at countries where geriatric medicine is still emerging^2^ and will be adapted to local contexts, the needs and assets of stakeholders and the pragmatic possibilities in appropriate settings.

This will be accomplished by the following tasks:

1. Description of the state-of-the-art of geriatric medicine education in involved countries
2. Identification of the global and local educational needs for developing clinical skills and competencies in geriatric medicine among medical doctors and allied healthcare professionals involved in the care of older patients across all healthcare services;
3. Reaching a consensus on core curricula in geriatric medicine for health care professionals (including medical doctors) by adapting global standards to fit the needs of diverse professional groups and pragmatic possibilities of local systems
4. Dissemination of the results on identified needs and proposed solutions to stakeholders, policy makers, and the public.

**Fig. 1:**
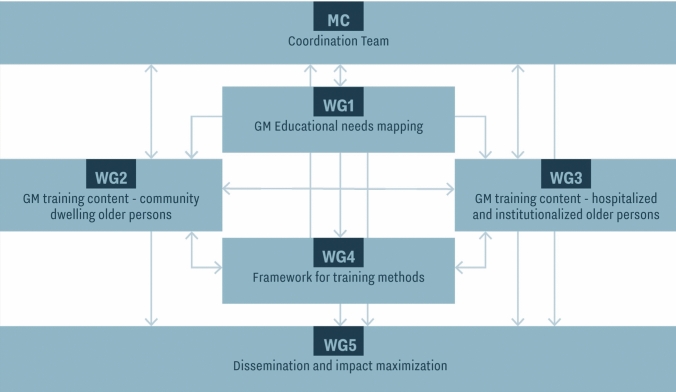
PROGRAMMING structure

To achieve these tasks, five Working Groups (WGs) were established to work collaboratively and with complementary roles. WG 1 focuses on tasks 1 and 2, gathering and organizing foundational data that will inform the activities of WGs 2, 3, and 4. These three groups address task 3 within distinct scopes: WG 2 focuses on ambulatory and home care settings; WG 3 on acute, sub-acute, and long-term care settings; and WG 4 on training methods. WG 5, dedicated to dissemination, communication, and maximizing impact, collaborates with all groups to ensure that findings and recommendations are effectively conveyed to relevant stakeholders. This coordinated approach allows each group to build upon and complement the work of the others, fostering a cohesive and impactful outcome (Fig. 1). Countries with well-established geriatric medicine systems will contribute with their experience and expertise in clinical and academic geriatrics. Nevertheless, even in these countries, we may observe significant gaps in geriatric culture and education; indeed, improvements are still to be made in the training of core geriatric competencies of the general workforce. Moreover, enhancing older adult care across Europe might be achieved by harmonizing the postgraduate curriculum in geriatric medicine among countries that already recognize it as an independent specialty [36]. A harmonized curriculum can standardize education, healthcare services, and care facilities for older adults [40]. Nevertheless, this effort falls outside the direct scope of PROGRAMMING and has been addressed by the Geriatric Medicine Section of the European Union of Medical Specialists (UEMS) for at least the past 20 years [55]. Their purpose is not to create a uniform curriculum, as national specifics are important, but to guarantee all countries include core competencies in their geriatric medicine curricula.

Another initiative aimed at this goal is the European Geriatric Medicine Specialty Exam, which seeks to standardize geriatricians’ knowledge across Europe and acknowledge their expertise. The first edition is scheduled for April 2025.

PROGRAMMING supports all initiatives aimed at improving the profession of geriatricians, as it will potentially advance geriatric medicine and healthcare for older adults [3].

PROGRAMMING aims to identify a pragmatic set of possibilities for continuous professional education in geriatric medicine, with the hope that these will be broadly endorsed by the scientific community of the medicine of aging and proposed to stakeholders and policymakers across Europe. The goal is to facilitate or even trigger change at each national level toward a minimum but substantial integration of principles of geriatric medicine in the attitudes and practices of practitioners. We expect to prepare the ground for more extended changes in countries where geriatric medicine is still emerging.

Furthermore, beyond concrete objectives of forming a core geriatric curriculum for non-geriatricians, the collaborative networking of PROGRAMMING aspires to achieve additional benefits for older adults, such as:

1. Raise awareness, promote, and acknowledge the added value of the specialized approach of geriatric medicine in the health and well-being of older people among health care professionals, policy makers, older people and the general public
2. Develop geriatric medicine -related literacy among health care professionals
3. Improve health literacy among older adults and empower them to advocate for the dissemination of geriatric medicine concepts and their right to better care wherever they live across Europe
4. Combat ageism among healthcare professionals, health policymakers and society

Widespread education in core concepts of geriatric medicine should facilitate the necessary cultural changes to eliminate misconceptions and foster age-tuned attitudes.

As we approach the end of the second year of the PROGRAMMING COST Action, we have successfully completed the tasks planned for the first half of the project. This includes the development of a state-of-the-art questionnaire on geriatric medicine education, training, and practices in Europe, as well as an assessment of the educational needs of more than 6,000 health professionals from over 70 countries and various healthcare settings, such as community facilities, hospitals, and long-term care facilities. Calls for researchers who are COST Action members to analyze different data sets are now open.

Since the beginning of the Action, a strong focus has been placed on teamwork and grant opportunities for individual and group activities that can foster GM. Many in-person international scientific meetings were held in countries where geriatric medicine still has significant opportunities for development, such as in Greece, Romania, North Macedonia, and Portugal. In these meetings, there was a deep commitment to engage national stakeholders to maximize the impact of the Action at the country level [2]. Communication and dissemination activities have been conducted from the very beginning of the Action, including various outputs published on social media channels and the website, as well as delivered individually by COST Action members, such as congress presentations and webinars [2]. The next steps include designing geriatric medicine curricula for different healthcare professionals in various healthcare settings, based on previously identified needs.

As of June 2024, 355 participants have joined PROGRAMMING, with different affiliations from 43 countries. Notably, a COST Action consists of an interdisciplinary network including researchers, players, and innovators from academia, industry, small and medium enterprises, public institutions, and other related organizations. This structure resembles the multidisciplinary team required in geriatric medicine clinical practice, research, and healthcare regulation.

The PROGRAMMING consortium is convinced that providing training for the existing workforce and influencing the attitudes of health professionals to enhance their awareness of basic principles in geriatric medicine can constitute a noteworthy and strategic approach to introducing geriatric medicine in countries where it is still in its initial phases. The WHO (2015) has previously stated that transforming the health workforce to ensure a sustainable and appropriately trained workforce is a priority action to provide better care to older persons and align healthcare systems to the needs of older people [24].

Improved knowledge, cultural changes, and ameliorated attitudes toward older age and the care of older people may also influence the design of new healthcare policies, strategically planning the development of clinical and social services adapted to the needs of older people. Building healthcare and social services tailored to the specific needs of older patients has been successfully implemented in various countries. This includes integrated care pathways in geriatric rehabilitation, orthogeriatric acute units, perioperative care services for older people undergoing surgery, frailty pathways in emergency departments, and oncogeriatrics models [56–58].

This progress is expected to be supported by relevant scientific societies and societal organizations and will need committed governments, through the implementation of supportive legislation, regulation, and financing programs, as advised by the WHO (2015) [24]. Engaging policy makers, whether directly linked to healthcare or not, is essential for consistency and effectiveness of age-related policies and trans-sectorial coherent plans. In addition, the PROGRAMMING COST Action can contribute toward 3 of the 17 sustainable development goals included in the *2030 Agenda for Sustainable Development* by United Nations, namely Good Health and Well Being (Goal 3), Quality Education (Goal 4) and Reduced Inequalities (Goal 10) [59].

As a large-scale, multinational initiative intending to engage diverse stakeholders and policymakers [60], the PROGRAMMING COST Action faces several challenges that require strategic attention during its 4-year period and beyond. One of the key challenges is ensuring long-term impact maximization, both during and after the Action. To achieve this, we must not only engage stakeholders effectively but also maintain their involvement beyond the lifespan of the project. Reaching and engaging national and international stakeholders—from healthcare professionals and policymakers to community organizations—requires targeted communication and disseminations strategies that highlight the relevance of geriatric medicine in their respective areas. For this purpose, professional communication and marketing skills may be helpful in developing a strategy for communication, dissemination, and impact maximization of the Action. It would be beneficial for COST Action members with this expertise to consider participating in these tasks. In addition, ensuring that the conceptual principles of geriatric medicine translate into real, effective changes in healthcare practices is another significant challenge. This will require pragmatic solutions, including aligning educational curricula with the clinical practice needs of each healthcare discipline at the national level. What is more, fostering collaboration among academic, clinical, and policy-making sectors will be essential. The integration of these principles into existing healthcare policies and services must be prioritized to ensure that geriatric medicine principles are recognized and effectively implemented. Finally, advancing the recognition of geriatric medicine as a distinct specialty is essential. Policies and services need to be designed not only to fulfill the core principles of geriatric medicine but also to meet the real-world needs of older adults. Despite these challenges, we are committed to identifying and implementing solutions that will ensure the long-term sustainability and effectiveness of the Action’s outcomes.

## A call for endorsement

**Fig. 2:**
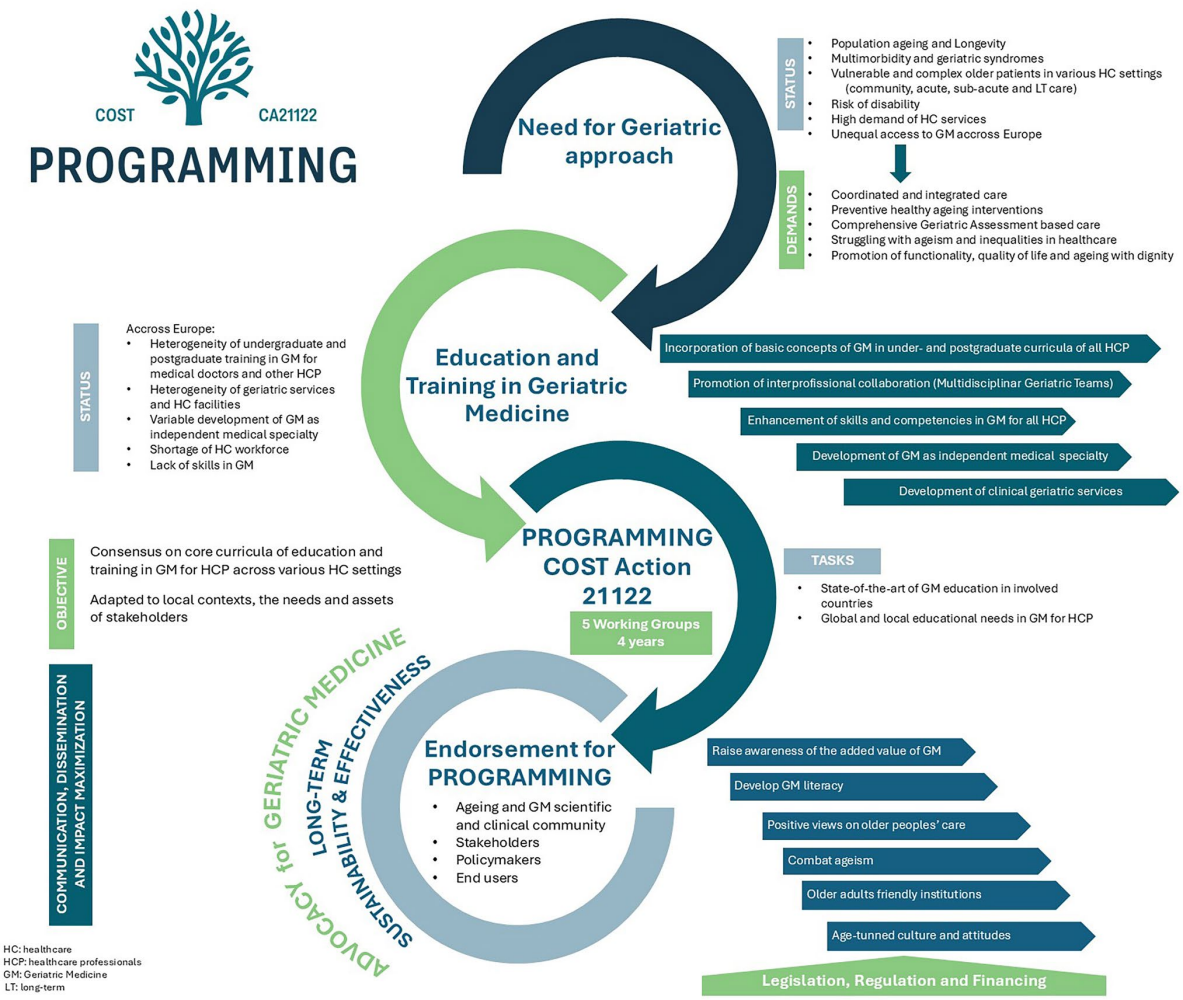
Graphical abstract: PROGRAMMING workflow

1. While the PROGRAMMING COST Action currently primarily comprises geriatricians, to achieve its main goals, a diverse network of participants across various disciplines is required. Among others, educators, healthcare professionals, policymakers, and representatives from scientific societies, as well as end users like older adults and caregivers, are invited to join the Action. On the scope of PROGRAMMING, a framework of stakeholders to be addressed to promote and develop geriatric medicine has already been discussed and published [60]. With the opportunity to join the COST Action available until 2026, engaging such a diverse range of members and expanding the Action’s network broadens its audience and reinforces its possibilities, widening and strengthening its impact. By expanding our network, we strengthen our capacity to collaborate and influence stakeholders, ensuring that our collective efforts resonate with both international and local policymakers.
2. The PROGRAMMING Consortium considers that shared objectives and clear, unified statements (Table), supported by leading societies in Geriatric Medicine and allied healthcare disciplines, will deliver a compelling message advocating for comprehensive and systematic education and training in geriatric medicine across Europe.
3. To foster these principles, this open call to endorse the document is available with a graphical abstract (Fig. 2), also for additional organizations to join, endorse, and disseminate locally and internationally at https://cost-programming.eu/ endorsement/.

## Conclusion

We aim to lay the groundwork for significant advancements in countries where geriatric medicine is still developing. A key component of this initiative will be the development of educational curricula designed to increase awareness and understanding of geriatric medicine among healthcare professionals. By equipping practitioners with essential knowledge and skills, we can foster a greater recognition of the importance of specialized care for older adults. This increased education and awareness will be crucial in influencing policymakers to prioritize the allocation of resources, including the establishment of more geriatric beds and specialized facilities. By demonstrating the necessity of enhanced geriatric medicine training and resources, we can advocate for structural changes that will improve the availability and quality of care for older individuals. Our educational initiatives will not only target non-specialist healthcare professionals but will also engage policymakers and stakeholders in discussions about the urgent need for systems.

## Footnotes

1 Belgium, Bulgaria, Croatia, Czech Republic, Germany, Denmark, Finland, France, Hungary, Iceland, Ireland, Italy, Lithuania, Luxembourg, Malta, Netherlands, Norway, Poland, Romania, Slovakia, Spain, Sweden, United Kingdom.

2 The Management Committee of PROGRAMMING CA has taken a conventional decision on the identification of 'Countries where Geriatric Medicine is still emerging', considering those that don’t have a postgraduate training program in Geriatric Medicine. In opposition, countries which have a postgraduate training program for obtaining a specialty title in Geriatrics are listed by Stuck and Masud [40].
